# Pan-eukaryotic distribution and deep homology of plant small secreted peptides and their receptors

**DOI:** 10.1016/j.isci.2026.115540

**Published:** 2026-03-30

**Authors:** Zhe Zhang, Songqing Yue, Fahu Yuan, Miaomiao Zhu

**Affiliations:** 1 Institute of Microalgae Synthetic Biology and Green Manufacturing, School of Life Sciences, Jianghan University, Wuhan 430056, China; 2 National Key Laboratory for Germplasm Innovation & Utilization of Horticultural Crops, Huazhong Agricultural University, Wuhan 430070, China; 3 State-owned Assets and Laboratory Management Office, Jianghan University, Wuhan 430056, China; 4 Innovation Center for Comprehensive Utilization of Food and Medicine Homologous Specialty Resources, School of Life Sciences, Jianghan University, Wuhan 430056, China; 5 Institute of Maternal and Children Health, Wuhan Children’s Hospital (Wuhan Maternal and Child Healthcare Hospital), Tongji Medical College, Huazhong University of Science and Technology, Wuhan 430015, China

**Keywords:** Evolutionary ecology, Evolutionary developmental biology, Plant evolution

## Abstract

Small secreted peptides (SSPs) are essential signaling molecules in plants, yet their deep evolutionary origins remained unclear. Here, we integrated sensitive homology searches with rigorous filtering to systematically identify SSP and leucine-rich repeats receptor-like kinase (LRR-RLK) homologs across the EukProt database. We recovered 59 high-confidence SSP homologs from diverse deeply branching eukaryotic supergroups, with protistan sequences consistently placed at the base of phylogenies. Parallel searches recovered 916 LRR-RLKs from non-vascular plant lineages, whereas the SSP-associated XI subfamily was largely confined to Archaeplastida. Co-occurrence of SSPs and LRR-RLKs in 6 lineages, together with bidirectional motif traceability revealing strong purifying selection on core residues, supports a model in which core components of SSP signaling emerged early in eukaryotic evolution and were vertically inherited, with subsequent lineage-specific diversification and co-option during streptophyte evolution. Our study provides a revised evolutionary framework for plant peptide signaling and a broadly applicable strategy for tracing ancient origins of lineage-specific innovations.

## Highlights

• We found 59 small secreted peptide (SSP) homologs in deeply branching eukaryotic supergroups
• Protistan SSP sequences are positioned at the base of plant phylogenies
• Fused leucine-rich repeats receptor-like kinases (LRR-RLKs) are widespread, and SSP receptor XI is Archaeplastida-specific
• Results support early eukaryotic origin and vertical inheritance of SSP signaling

## Graphical abstract

**Figure:**
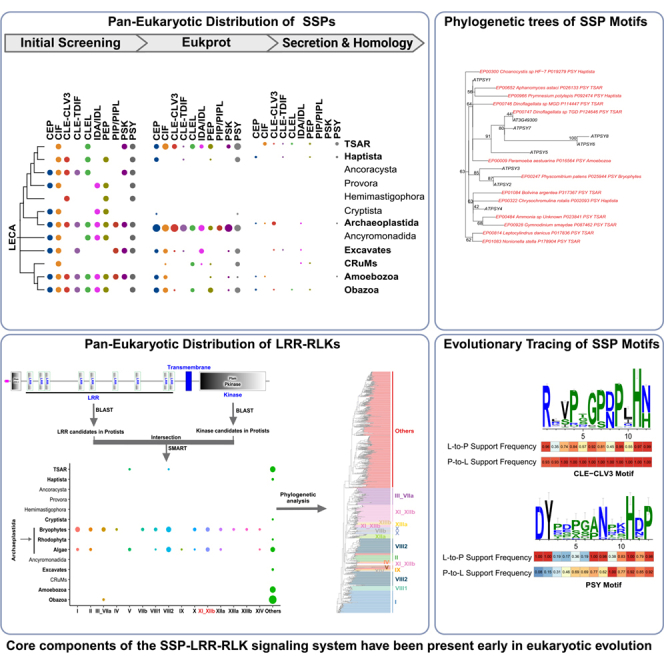

## Introduction

Plant small secreted peptides (SSPs) are typically short peptides or proteins—often less than 250 amino acids—that are released into the extracellular space via conventional or unconventional secretion pathways. These peptides function as critical mediators in a wide array of processes such as growth regulation, developmental signaling, defense responses, and symbiotic interactions with beneficial microbes.^1^^,^^2^^,^^3^^,^^4^^,^^5^^,^^6^^,^^7^^,^^8^ Small post-translationally modified peptides (PTMPs) are an important class of SSP peptides, including very small mature peptides that often undergo essential post-translational modifications such as hydroxylation, sulfation, or glycosylation; these modifications are critical for their receptor-binding activity and stability.^9^^,^^10^^,^^11^^,^^12^^,^^13^^,^^14^ Additionally, mature PTMPs have been reported to bind the corresponding receptor leucine-rich repeat receptor-like kinases (LRR-RLKs), which suggests the homology of mature PTMP.^12^^,^^14^ However, due to the short length and relatively low sequence identity of mature PTMP amino acids (often <20 amino acids), it is difficult to conduct phylogenetic analysis on them. Interestingly, the receptors of most PTMPs are located in LRR-RLK subfamily XI. Thus, 9 families SSPs whose receptors in of LRR-RLKs XI, including CLE(CLAVATA3/EMBRYO SURROUNDING REGION-related^15^), PIP/PIPL(PAMP-INDUCED SECRETED PEPTIDE, PIP-LIKE^16^), CEP(C-TERMINALLY ENCODED PEPTIDEs^17^), IDA/IDL(INFLORESCENCE DEFICIENT IN ABSCISSION, IDA-LIKE^18^^,^^19^), CLEL(CLE-LIKE/ROOT GROWTH FACTOR/GOLVEN^20^), PSY(PLANT PEPTIDE CONTAINING SULFATED TYROSINE^21^), CIF(CASPARIAN STRIP INTEGRITY FACTORs^22^), PEP(PLANT ELICITOR PEPTIDES^23^), and PSK(PHYTOSULFOKINE^24^), were chosen for phylogenetic analysis in this study.

Previous studies had identified SSPs in moss plants.^11^^,^^12^^,^^25^ Coincidentally, mosses were the surviving representatives of early differentiated terrestrial plant lineages, so some studies speculated that SSPs may have originated from a common ancestor of terrestrial plants.^13^^,^^14^ Although SSP has not been directly identified in native organisms, several comparative genomics studies have shown that the origin of certain components of hormones and stress signaling pathways in terrestrial plants can be traced back to algae. It means that SSP and its receptors are likely to also appear in algae and even protists.^12^^,^^26^^,^^27^ However, the identification of SSP peptides is often limited by genome quality, genome assembly strategies, and gene annotation methods.

In this study, we developed an integrated computational pipeline that combines transcriptome-sensitive homology searches with rigorous, multistep filtering, including signal peptide prediction, transmembrane topology validation, Pfam domain annotation, and HMMER-based profile searches. This approach allowed us to overcome the long-standing challenges of annotating short, rapidly evolving SSP genes in non-model eukaryotes. Applying this strategy to the comprehensive EukProt database, we identified high-confidence SSP homologs across multiple deeply branching eukaryotic supergroups, with several families such as PSY, CEP, and CIF exhibiting broad and phylogenetically structured distributions. Parallel searches for their cognate receptors recovered hundreds of structurally intact, fused LRR-RLK kinases from diverse eukaryotic lineages. Phylogenetic analyses revealed that most non-plant receptors occupy deeply divergent basal positions, whereas the SSP-associated XI subfamily is largely confined to Archaeplastida. The consistent placement of protistan SSP sequences at the base of the land plant green algal clade, together with the co-occurrence of SSP ligands and complete LRR-RLKs in multiple non-plant lineages, supports a model in which core components of the SSP signaling system emerged early in eukaryotic evolution. These components were subsequently inherited, differentially retained, and in the streptophyte lineage further co-opted and diversified to underpin the complex peptide signaling networks of land plants. Our work not only revises the evolutionary history of a key plant communication module but also provides a methodological framework broadly applicable to uncovering the ancient origins of other traits currently considered lineage-specific innovations.

## Results

### Pan-eukaryotic distribution of small secreted peptide (SSP) families

**Figure 1:**
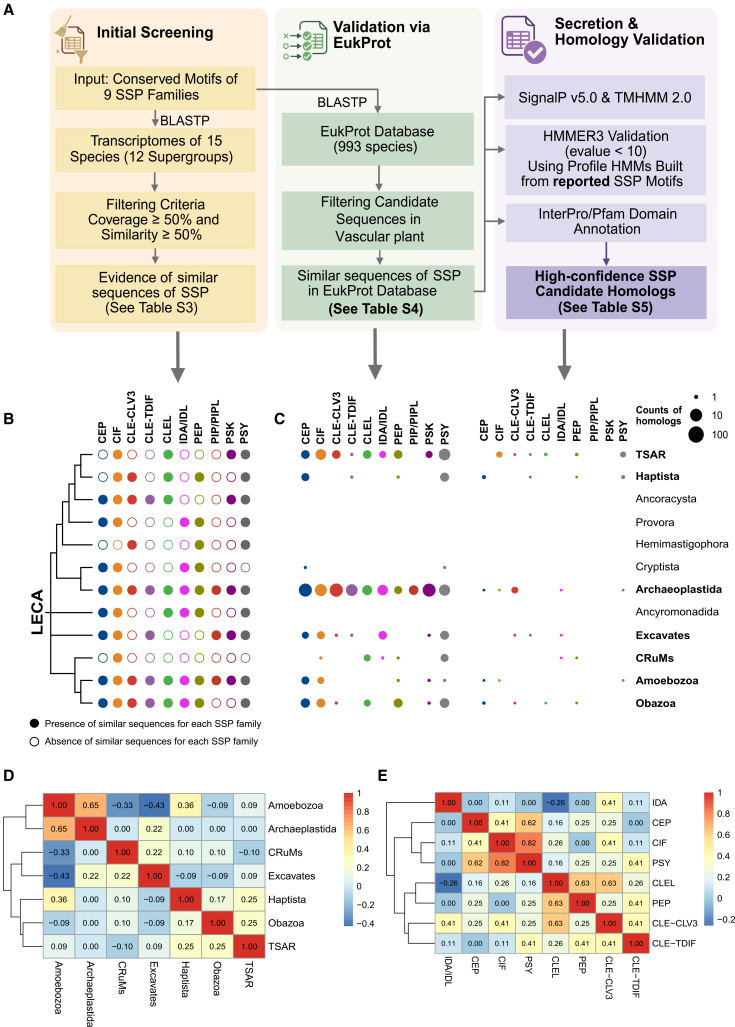
Distribution of candidate SSP homologs sequences in protists (A) Identification process of candidate SSP homologs in the Eukprot database. (B) Presence (filled circle) or absence (empty circle) of homologs for each SSP family. (C) Presence (filled circle) and counts of candidates/homologs for each SSP family. (D and E) Heatmap shows pairwise Pearson’s correlation coefficients calculated based on the presence/absence profiles of SSP families across eukaryotic lineages (D) and SSP families (E), statistical significance was not detected (*P* threshold = 0.05) for any of the pairwise correlations in (D) or (E).

This study investigated the origin of plant SSP families, including CLE, PIP/PIPL, CEP, IDA/IDL, CLEL, PSY, CIF, PEP, and PSK (Table S1). Using BLAST, sequences with similarity to the motifs of these SSP families were identified in RNA-seq data from 15 species spanning 12 eukaryotic supergroups: TSAR, Haptista, Ancoracysta, Provora, Hemimastigophora, Cryptista, Archaeoplastida, Ancyromonadida, Excavates, CRuMs, Amoebozoa, and Obazoa (Table 1, Figures 1A and 1B). Similar sequences for at least one SSP family were detected in all 12 supergroups. Notably, similarity to all 9 target SSP families was found in the unicellular green alga *Chlamydomonas reinhardtii* (Archaeoplastida). These results indicate that SSP-like sequences are present in diverse lineages far beyond land plants, including algae and various protists (Table S2). The discovery of these sequences in deeply branching eukaryotic supergroups provides initial support for the hypothesis that the ancestral components of SSP signaling may trace back to the last eukaryotic common ancestor (LECA). Definitive confirmation of homology and an LECA origin requires further phylogenetic and functional validation, as undertaken in subsequent analyses.

**Table 1:** SRA accession code of the eukaryotic supergroup in this study

Classification	Species	SRA accesson
TSAR	*Tetrahymena thermophila*	DRR440460
	*Telonema subtile*	SRR7371267
Haptista	*Raphidiophrys heterophryoidea*	SRX1155946
Ancoracysta	*Ancoracysta twistia*	SRX3270723
Provora	*Nibbleromonas arcticus*	SRX16844583
Hemimastigophora	*Hemimastix kukwesijik*	SRX3182827
Cryptista	*Palpitomonas bilix*	SRX549024
Archaeoplastida	*Chlamydomonas reinhardtii*	DRX019850
Ancyromonadida	*Ancyromonas sigmoides*	SRX3153021
	*Fabomonas mesopelagica*	SRX19309166
Excavates	*Trichomonas vaginalis*	SRR2007500
CRuMs	*Rigifila ramosa*	SRX3153022
Amoebozoa	*Dictyostelium discoideum*	DRR035699
Obazoa	*Amastigomonas*	SRX1155951
	*Saccharomyces cerevisiae*	DRR023778

Then, following the SSP identification pipeline described above (Figure 1A), we performed a systematic search for SSP homologs within the EukProt database. Initial BLAST searches were followed by a multi-step filtering procedure to ensure the reliability of candidate sequences: (1) removal of sequences from vascular plants to retain only those from bryophytes, algae, and protists; (2) validation using HMMER3 with family-specific profile hidden Markov models (e-value <10) to capture divergent yet genuine homologs with high sensitivity; and (3) elimination of false positives via InterPro/Pfam domain annotation, excluding sequences confidently annotated to protein families unrelated to SSPs. Through this rigorous pipeline, we identified strong candidate homologs of SSP families in 7 major eukaryotic lineages: TSAR, Haptista, Archaeoplastida, Excavates, CRuMs, Amoebozoa, and Obazoa (Figure 1B). Their identification across these diverse supergroups reinforces the deep and widespread distribution of SSP-like signaling components in the eukaryotic domain, providing a refined dataset for subsequent phylogenetic validation of homology.

Furthermore, to assess the robustness of our phylogenetic inferences and to partially overcome the signal limitations imposed by short conserved motifs (Table S4), we performed independent phylogenetic analyses using both SSP conserved motifs and full-length precursor protein sequences (maximum likelihood, LG model, 1,000 bootstrap replicates). For major, widely distributed families such as PSY, CEP, IDA/IDL, and CIF, the two analytical strategies yielded highly congruent core topologies: Homologous sequences from multiple protistan supergroups (e.g., TSAR, Excavates, and Haptista) consistently tended to be positioned near the base of the major clade formed by land plants (Figures S1 and S2). Although the use of full-length sequences moderately improved support values for some nodes, the overall length of SSP precursors remains limited (typically 80–150 amino acids), and thus phylogenetic resolution over deep evolutionary timescales is still constrained. The concordant phylogenetic patterns recovered from two independent datasets and analytical approaches substantially strengthen our confidence in the inferred core evolutionary relationships. Taken together, the observed phylogenetic topology is consistent with a model of vertical inheritance and provides supportive evidence for the hypothesis that core components of the SSP signaling system were already present in the LECA, although it does not uniquely exclude alternative evolutionary scenarios (e.g., convergent evolution or horizontal gene transfer (HGT)).

### An internal evolutionary relationship in SSPs

The phylogenetic distribution of the 9 SSP families and their distinct subclades across 12 major eukaryotic lineages revealed considerable heterogeneity (Figure 1C). Based on our stringent filtering pipeline, the PSY, CEP, PEP, and CLE-CLV3 subclades were each detected in 4 lineages. The CIF, CLE-TDIF subclade, and IDA/IDL families were identified in three lineages. CLEL showed the most restricted distribution among the detected families, found only in Obazoa and TSAR (two lineages). In contrast, the PIP/PIPL and PSK families were not detected in any non-vascular plant lineage; consistent with previous reports, they appear to be vascular plant-specific innovations.

This differential distribution pattern suggests distinct evolutionary trajectories. Families with relatively broad presence across multiple deeply branching lineages (e.g., PSY, CEP, PEP, and the CLE-CLV3 subclade) likely represent ancient components of the eukaryotic signaling toolkit. The intermediate but consistent occurrence of CIF and CLE-TDIF further supports their roles as fundamentally conserved modules that diversified in specific lineages. The contrasting distribution of the two CLE subclades (CLV3-type versus TDIF-type) highlights subfunctionalization events that occurred early in eukaryotic evolution. Conversely, the complete absence of PIP/PIPL and PSK in all surveyed non-vascular lineages reinforces their interpretation as more recent innovations restricted to the vascular plant lineage.

To explore the evolutionary relationships underlying the differential distribution of SSP families, we performed pairwise Pearson’s correlation analyses based on the binary presence/absence data across seven major eukaryotic lineages and across nine SSP families/subclades (Figures 1C and 1D). Lineage-level correlations were generally weak (|r| < 0.3), consistent with extensive lineage-specific gene retention and loss after ancient origin. A moderate positive correlation was observed only between Archaeplastida and Amoebozoa (Pearson’s r = 0.65, *p* = 0.12), suggesting that these two deeply diverged lineages share a subset of conserved SSP families (e.g., PSY and CEP). While SSP-family correlations revealed several strong positive associations. The highest correlation occurred between PSY and CIF (Pearson’s r = 0.82, *p* = 0.052), followed by PSY-CEP (Pearson’s r = 0.63, *p* = 0.35), CLE-CLV3–CLEL (Pearson’s r = 0.63, *p* = 0.20), and CLEL-PEP (Pearson’s r = 0.63, *p* = 0.20). These pairs tend to co-occur or be co-absent across lineages, implying potential functional coupling or shared evolutionary constraints. In contrast, IDA/IDL showed weak or negative correlations with most families, indicating a more independent distribution.

We next investigated whether a family’s broad phylogenetic distribution predicted its propensity for gene expansion within a derived lineage. Statistical analysis revealed no significant correlation between the number of protistan groups harboring an SSP family and its gene copy number in *Arabidopsis thaliana* (Pearson’s r = 0.32, *p* = 0.44). This lack of correlation is strikingly illustrated by comparing the CIF and CLE-CLV3 families. Despite both CIF and the CLE-CLV3/CLE-TDIF subfamilies being detected in 3–4 eukaryotic lineages, their copy numbers in *Arabidopsis* are starkly different. The CLE-CLV3 family has undergone massive expansion to at least 28 members, whereas the CIF family remains small with only 4 members. This demonstrates that deep evolutionary conservation does not necessarily predispose a family to high copy number in a specific descendant lineage, and highlights that recent, lineage-specific expansion is a major driver of SSP repertoire diversity.

### Distribution and phylogenetic placement of LRR-RLK receptors across eukaryotes

**Figure 2:**
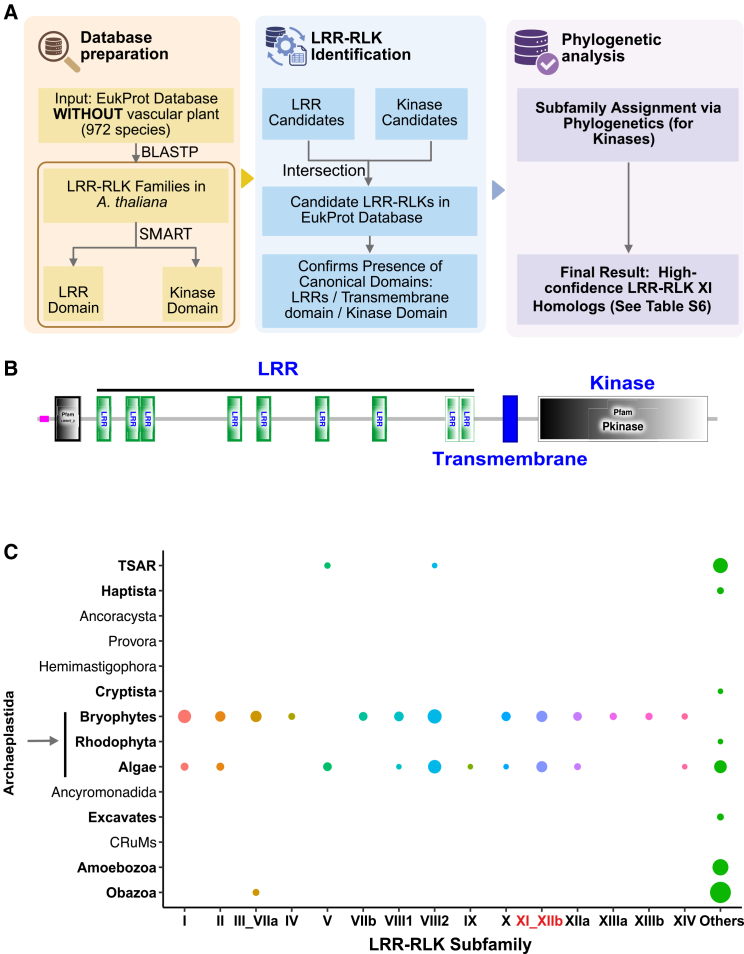
Distribution of candidate LRR-RLK XI homologs sequences in protists (A) Identification process of candidate LRR-RLK XI homologs in the Eukprot database. (B) Typical protein domain of LRR-RLK XI. (C) Presence (filled circle) and counts of candidate homologs for each LRR-RLK subfamily.

To investigate whether the cognate receptor components of SSP signaling systems exhibit a similarly broad phylogenetic distribution, we performed a systematic search for LRR-RLK homologs across the EukProt database using an integrated strategy combining domain-specific BLAST searches (with LRR and kinase domains as independent databases), reciprocal hit filtering, and SMART-based domain architecture validation. Only sequences containing a complete, N-to-C terminal LRR-transmembrane-kinase tripartite domain organization were retained as high-confidence LRR-RLK candidates. These were subsequently subjected to phylogenetic analysis together with reference LRR-RLK subfamily members from *Arabidopsis* (Figures 2A and 2B, Table S5).

Our analysis recovered 916 high-confidence LRR-RLK sequences distributed across multiple eukaryotic lineages, including Archaeplastida (including Algae, Bryophytes, Rhodophyta), Amoebozoa, Obazoa, TSAR, Excavates, Haptista, and Cryptista (Figure 2C). Notably, the phylogenetic classification revealed a striking pattern: The majority of LRR-RLK candidates from non-Archaeplastida lineages (e.g., Amoebozoa, Obazoa, and TSAR) fell into a large, deeply branching “others” category positioned near the base of the LRR-RLK tree, rather than nesting within any of the established *Arabidopsis* subfamilies (Figures 2C and S3, Table S5). In contrast, the known receptors for SSPs were exclusively confined to Archaeplastida (Algae and Bryophytes), with only sporadic occurrences elsewhere (Figures 2C and S3).

This distribution pattern bears notable parallels to our SSP findings. Just as ancient, widely distributed SSP families (e.g., PSY and CEP) were identified across multiple protistan lineages, we now demonstrate that structurally intact, fused LRR-RLKs are also present in these same lineages. However, the receptor sequences recovered from non-Archaeplastida eukaryotes predominantly represent deeply divergent, phylogenetically basal forms, suggesting that while the core architecture of LRR-RLK was established early in eukaryotic evolution, the specific receptor subfamilies later co-opted for SSP signaling in land plants (particularly subfamily XI) underwent major diversification and expansion primarily within the Archaeplastida.

The co-occurrence of SSP ligands and complete LRR-RLK receptors in several non-plant lineages (e.g., Amoebozoa and TSAR) is consistent with the possibility that a primitive SSP-responsive signaling module emerged early in eukaryotic evolution and was subsequently inherited by diverse lineages. However, the phylogenetic distinctiveness of most non-Archaeplastida receptors implies that they may recognize different ligands or function in alternative physiological contexts, with the plant-type SSP-LRR-RLK XI signaling pair representing a specialized, highly conserved sub-system that was refined during streptophyte evolution and land plant terrestrialization.

### Evolutionary tracing of land plants SSP motifs to protistan ancestors

**Figure 3:**
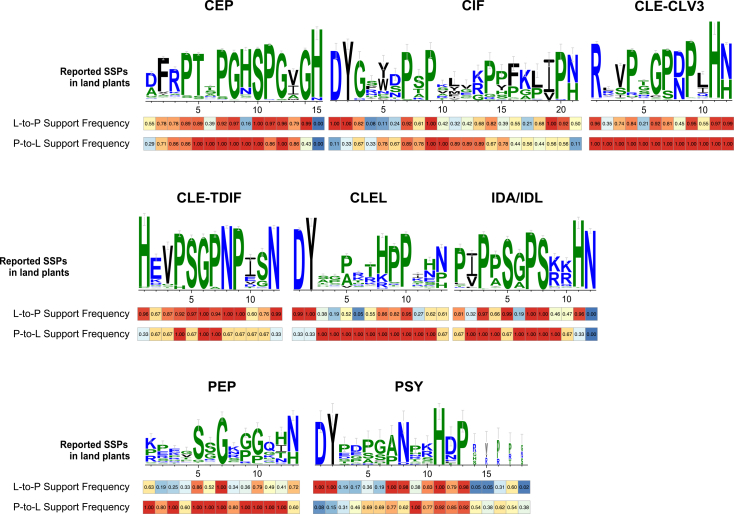
Evolutionary land plant support frequency, and land plant-to-protistan traceability frequency of each site of the conserved motif of each SSP family (Top) Sequence logo of conserved motifs from reported plant SSP families, showing position-specific information content (bits). Error bars indicate the standard deviation estimated from 1,000 bootstrap resamples, reflecting uncertainty in the information content measurement. (Bottom) Protistan-to-land plant support frequency, and land plant-to-protistan traceability frequency of each site of conserved motif of each SSP family.

To accurately evaluate the evolutionary constraints on functional motifs, we calculated the percentage of supported sites considering only the SSP families that were actually identified in each supergroup. The bidirectional frequency analysis of supported sites provided robust, quantitative evidence for deep homology and revealed distinct evolutionary dynamics across the SSP motif positions (Figure 3).

For the core functional motifs, an overwhelming majority of key sites showed exceptionally high bidirectional support. For instance, in the CEP families, over 70% (11 out of 15) of plant reference motif residues had traceability frequencies (L→P) exceeding 70%, meaning their amino acid states are predominantly found in protistan homologs. Concurrently, the corresponding residues in protists showed high conservation frequencies (P→L) in plants, often above 85% (11 out of 15). This strong reciprocal conservation signifies that these core positions have been under intense purifying selection since before the divergence of major eukaryotic supergroups (Figure 3).

Furthermore, the analysis delineated positions with asymmetric evolutionary patterns. A subset of sites exhibited high plant-to-protistan traceability (L→P) but moderate protistan-to-plant conservation (P→L). This pattern suggests that while the ancestral sequence space permitted some variation at these positions, a specific amino acid state became fixed and optimized within the land plant lineage. Conversely, a few sites showed the opposite asymmetry, potentially indicating lineage-specific innovations in certain protistan groups, such as CLEL and IDA/IDL. The application of this bidirectional frequency metric thus not only solidifies the evidence for the ancient origin of SSPs but also refines our understanding of the evolutionary landscape, distinguishing between immutably conserved cores and regions that have undergone functional tuning during eukaryotic diversification.

## Discussion

### Alternative evolutionary scenarios: Convergent evolution and horizontal gene transfer

The hypothesis that similar SSP motifs arose independently in distantly related eukaryotes through convergent evolution is difficult to reconcile with several lines of evidence:

Complexity and Co-occurrence: Convergence might explain the independent origin of a single, short motif under similar selective pressures. However, it is statistically improbable for the entire suite of observed features—including (1) multiple distinct motif families (e.g., CLE-CLV3, PSY, CEP, and CIF), (2) some of them association with predicted N-terminal secretion signals, (3) their conserved C-terminal position within precursors, and (4) their specific co-occurrence in the same lineages with the kinase domains of their putative LRR-RLK receptors. This coordinated set of traits is more readily explained by the inheritance and diversification of an integrated system from a common ancestor.

Phylogenetic Signal: Our phylogenetic analyses show that protistan SSPs are not randomly related but are nested as orthologs within well-defined plant SSP families (Figures S1 and S2). Convergent evolution would not produce this consistent, hierarchy-preserving phylogenetic pattern; it would instead result in sequences from distantly related protists grouping together based on motif similarity, irrespective of species phylogeny, which we do not observe.

While HGT can distribute genes across lineages, it is an unlikely primary mechanism for the observed pan-eukaryotic distribution of the SSP signaling system:

Pattern of Distribution: HGT events are typically sporadic and patchy. The widespread, yet phylogenetically structured, the presence of SSP families across nearly all major eukaryotic supergroups (e.g., PSY in 8/8 protist groups) suggests a pattern of vertical inheritance with lineage-specific losses, rather than a reticulate network of recent transfers. A scenario requiring numerous, independent HGT events into diverse eukaryotic lineages is less parsimonious than a single origin in LECA.

Co-transfer Barrier: For HGT to explain a functional signaling module, it would require the simultaneous transfer and functional integration of both the ligand (SSP) and its cognate receptor (LRR-RLK XI) genes—a highly complex and unlikely event. Our data show these components co-occur in multiple deep-branching lineages, further supporting their shared vertical history.

Receptor Architecture Evolution: Our finding that the LRR and kinase domains are often dissociated in early-branching eukaryotes argues against a recent, plant-like fused receptor being transferred. Instead, it points to an ancient, flexible ancestral state that evolved vertically.

While neither convergent evolution nor HGT can be definitively excluded in individual cases, the accumulated evidence, including broad phylogenetic distribution, conserved domain architecture, consistent phylogenetic placement of protistan sequences, and coordinated occurrence of ligands and receptor components, is most consistent with a model of vertical inheritance from an ancestral signaling system present early in eukaryotic evolution. This interpretation provides a useful framework for future functional studies aimed at understanding how this ancient molecular toolkit was repurposed for diverse cellular communication tasks across the eukaryotic Tree of Life.

### Tracing the evolutionary dynamics of SSP functional motifs

The bidirectional frequency analysis of conserved SSP motifs provides a quantitative window into the evolutionary forces that have shaped these critical signaling components over deep time. The exceptionally high reciprocal conservation observed at core functional sites—as exemplified by the CEP family, where over 70% of key plant residues are traceable to protistan homologs (L→P >80%) and vice versa (P→L >85%)—strongly argues for their origin in a common ancestral sequence. This pattern is most parsimoniously explained by intense and continuous purifying selection acting to maintain essential biochemical or structural properties since before the radiation of major eukaryotic supergroups. The statistical robustness of this bidirectional signal makes convergent evolution an unlikely explanation, thereby lending substantial weight to the hypothesis that a functional SSP signaling module was present in the early eukaryotes.

Furthermore, the identification of sites with asymmetric evolutionary patterns offers nuanced insights into lineage-specific adaptation. The subset of positions with high plant-to-protistan traceability but only moderate reciprocal conservation suggests a scenario where an ancestral, somewhat flexible residue was later optimized and fixed within the land plant lineage, possibly to refine interaction specificity or signaling strength in the context of multicellular complexity. Conversely, the opposite asymmetry observed in some protistan groups (e.g., CLEL and IDA/IDL) highlights potential innovations unique to those lineages. This delineation between a universally constrained core and malleable peripheral sites illustrates how a deeply ancient system could provide a stable scaffold upon which functional diversification occurred. It underscores that the evolutionary history of SSPs is not one of static preservation but involves both remarkable conservation of a central mechanism and strategic tuning at specific positions, enabling the system to adapt to the diverse physiological contexts encountered across the eukaryotic Tree of Life.

### Analytical framework and evolutionary insights

A key reason previous studies failed to identify SSPs in protists lies in methodological limitations. Standard genome annotation pipelines are notoriously biased against predicting small, non-canonical genes that encode SSPs. In our study, direct screening of transcriptomic data revealed the presence of SSP-like sequences in protists. Furthermore, a comprehensive search of the eukaryotic database Eukprot led to the identification of candidate SSP homologs and their receptors. This approach allowed us to find expressed genes regardless of their annotation status in genomic databases. This methodological shift is not merely a technical detail; it represents a powerful paradigm for evolutionary “origin tracing” studies. It suggests that the genetic ancestry of many other putative lineage-specific traits—be they transcription factors, metabolic enzymes, or other signaling components—may be far deeper than currently recognized. By applying this sensitive, motif-centric approach to transcriptomic data from diverse, non-model eukaryotes, we can expect to uncover the deep evolutionary roots of other complex biological systems, ultimately leading to a more accurate and detailed understanding of ancestral gene repertoires and the evolutionary history of early eukaryotes.

### Limitations of the study

Several limitations of this study should be acknowledged. First, our analyses rely primarily on transcriptome-derived data from the EukProt database, which may not represent complete gene repertoires and could include assembly artifacts or contamination. Second, the short length and low sequence complexity of SSP mature motifs inherently limit phylogenetic resolution at deep evolutionary timescales, making it difficult to confidently resolve relationships among deeply divergent lineages. Third, while we applied stringent criteria to ensure candidate sequences share functional features with plant SSPs (e.g., signal peptides and conserved motif architecture), we cannot exclude the possibility that some identified homologs represent convergent evolution rather than true orthology. Fourth, our inferences of purifying selection based on bidirectional traceability, while statistically robust, remain indirect and do not demonstrate conserved biological function. Finally, the co-occurrence of SSP-like ligands and LRR-RLK receptors in non-plant lineages suggests, but does not prove, the existence of functional signaling modules; experimental validation is required to determine whether these components interact and what physiological roles they may play. Future studies incorporating genomic data from additional key lineages, together with functional characterization, will be essential to test and refine the evolutionary framework proposed here.

## Resource availability

### Lead contact

Requests for further information and resources should be directed to, and will be fulfilled by, the lead contact, Miaomiao Zhu (zhumiaomiao@zgwhfe.com).

### Materials availability

This study did not generate new unique reagents.

### Data and code availability

• All final data generated or analyzed during this study (e.g., candidate SSP and LRR-RLK homologs, phylogenetic trees, and traceability scores) are included in this published article and its supplementary information files.
• The custom R code for calculating evolutionary traceability scores, along with the input alignments required to reproduce the analysis, has been deposited at GitHub: https://github.com/Busydog1990/evolutionary_traceability.
• All other software used in this study (BLAST, SMART, HMMER, SignalP, TMHMM, IQ-TREE, and so forth) is publicly available as described in the Methods section.
• Any additional information required to reanalyze the data reported in this paper is available from the lead contact upon request.

## Acknowledgments

We are grateful to Prof. Bo Zheng for his guidance on this research. This work was supported by the 10.13039/100007219Wuhan Natural Science Foundation-Exploration Program (Morning Light Program) (2024040801020375), the Funding for Scientific Research Projects from Wuhan Children’s Hospital (2025FEBSJJ006), the 10.13039/501100003819Natural Science Foundation of Hubei Province of China (2023AFB345), and the 10.13039/501100012613University-Industry Collaborative Education Program (22097040141133, 231104794215900). This project is supported by the Intelligent Computing Center of Jianghan University.

## Author contributions

M. Z., Z. Z., and F. Y designed the research. Z. Z. and S. Y. collected and analyzed the data. Z.Z. drafted the manuscript. M. Z. and F. Y. revised the article. All authors read and approved the final manuscript.

## Declaration of interests

The authors declare that they have no competing interests.

## STAR★Methods

### Key resources table

**Table:** 

REAGENT or RESOURCE	SOURCE	IDENTIFIER
**Deposited data**		

scRNAseq of *Tetrahymena thermophila*	SRA	DRR440460
RNA-Seq of *Telonema subtile*	SRA	SRR7371267
*Raphidiophrys heterophryoidea* RNA-seq	SRA	SRX1155946
The transcriptome of *Ancoracysta twista*	SRA	SRX3270723
RNA-Seq of *Nibbleromonas arcticus*	SRA	SRX16844583
raw RNA-Seq data of *Hemimastix kukwesjijk*	SRA	SRX3182827
Whole transcriptome sequencing of *Palpitomonas bilix* NIES-2562 - MMETSP0780_2	SRA	SRX549024
RNA-seq for Chlamydomonas tar1-1 mutant	SRA	DRX019850
Ancyromonas sigmoides CCAP 1958/3 transcriptome	SRA	SRX3153021
RNA-seq of *Fabomonas tropica*	SRA	SRX19309166
*Trichomonas vaginalis* Genome sequencing	SRA	SRR2007500
*Rigifila ramosa* CCAP 1967/1 transcriptome	SRA	SRX3153022
RNA-Seq of *Dictyostelium discoideum*	SRA	DRR035699
*Amastigomonas* sp. RNA-seq	SRA	SRX1155951
RNA-Seq of *Saccharomyces cerevisiae*	SRA	DRR023778

**Software and algorithms**		

BLAST	Altschul et al.^28^	https://blast.ncbi.nlm.nih.gov/Blast.cgi
EukProt	Richter et al.^29^	https://evocellbio.com/eukprot/
SignalP	Teufel et al.^30^	https://services.healthtech.dtu.dk/services/SignalP-6.0/
TMHMM	Krogh et al.^31^	https://services.healthtech.dtu.dk/services/TMHMM-2.0/
HMMER	Eddy SR^32^	http://www.hmmer.org/
InterPro	Blum et al.^33^	https://www.ebi.ac.uk/interpro/
Pfam	Paysan-Lafosse et al.^34^	https://www.ebi.ac.uk/interpro/
R packages msa	Bodenhofer et al.^35^	http://www.bioinf.jku.at/software/msa/
SMART	Letunic I and Bork P^36^	https://smart.embl.de
IQ-TREE	Wong et al.^37^	https://iqtree.github.io/
Clustal Omega	Sievers et al.^38^	https://www.ebi.ac.uk/jdispatcher/msa/clustalo
Weblogo 3	Crooks et al.^39^	https://weblogo.threeplusone.com/
Evolutionary Traceability Calculation	This study	https://github.com/Busydog1990/evolutionary_traceability

### Experimental model and study participant details

This study did not involve any experimental models (such as animals, plants, microbe strains, cell lines, or primary cell cultures) or human participants. All analyses were performed using publicly available data and software, as described in the Methods section.

### Method details

#### Identification of small secreted proteins (SSPs)

To systematically identify small secreted proteins (SSPs) across a broad range of eukaryotes, we employed a progressive, multi-step computational pipeline that integrated initial screening of transcriptome data with validation against a high-quality protein database.

First, to conduct a preliminary exploration of SSP distribution in deeply branching eukaryotic lineages, we performed an initial BLAST search^28^ for 9 major SSP families using transcriptome data from 15 species representing 12 eukaryotic supergroups (Table S1). The query sequences consisted of reported amino acid sequences of 9 SSP families (CLE,^12^^,^^40^ PIP/PIPL,^41^^,^^42^^,^^43^ CEP,^15^^,^^44^ IDA/IDL,^12^^,^^19^^,^^40^ CLEL,^12^^,^^45^ PSY,^12^^,^^21^^,^^46^ CIF,^12^^,^^22^ PEP,^24^^,^^47^ and PSK^12^^,^^48^^,^^49^) and LRR-RLKs from representative land plants (Table S2). Conserved motifs from each SSP family were used as queries in a permissive BLASTP search (E-value cutoff ≤10,000). Matches were subsequently filtered to retain only those where the alignment covered at least 50% of the query motif length and shared a minimum sequence similarity of 50% (Table S3). This step aimed to demonstrate the presence of sequences bearing similarity to SSP family motifs in the relevant transcriptomes, not to directly establish homology. Due to the lack of extensive assembly and curation of the raw transcriptome data used, it was challenging to reliably obtain full-length coding sequences for candidate peptides from this source for further analysis.

To obtain more reliable homologous sequences, we shifted our focus to the assembled EukProt database.^29^ Using the same set of SSP family conserved motifs as queries, we performed a BLASTP search against the protein sequences of 993 species in EukProt, applying identical coverage and similarity filters (coverage ≥50%, similarity ≥50%) (Table S4). To further validate that the candidate sequences exhibited characteristics of secreted proteins, we subjected all filtered candidates to signal peptide prediction using SignalP v5.0.^30^ We also performed transmembrane helix prediction using TMHMM 2.0.^31^ Since transmembrane domains and signal peptides share similar hydrophobic sequence features, TMHMM predictions can be ambiguous and were therefore treated as supportive references rather than strict exclusion criteria. Concurrently, we calculated the alignment position of the SSP conserved motif within each candidate sequence. As the mature, active SSP motif is typically located within the C-terminal region of the precursor protein, this positional information served as an important auxiliary criterion. It is important to note that sequences in EukProt are also derived from transcriptome assemblies and are not guaranteed to be full-length. Therefore, the SignalP prediction results, TMHMM predictions, and motif alignment positions were all treated as key supporting indicators in our study, not as absolute determinants.

Then, to enhance the rigor of our identification, we utilized HMMER^32^ to build profile hidden Markov models (Profile HMMs) based on the conserved motifs of each reported SSPs. All candidate sequences were searched against these models, and those with an e-value ≤10 were retained. These candidates were further subjected to InterPro/Pfam domain annotation (https://www.ebi.ac.uk/interpro/),^33^^,^^34^ any sequence confidently annotated to a protein family unrelated to small secreted peptides was removed.

The final set of SSP candidate homologs that passed this multi-step filtering and validation pipeline is compiled in Table S4.

All protein sequences were analyzed in the R statistical computing environment. Multiple sequence alignment (MSA) was performed using the ClustalW algorithm implemented in the msa R package.^35^

Phylogenetic trees were constructed using the ape package. The amino acid distance matrix was calculated, and the phylogenetic trees were inferred using the Maximum Likelihood, LG model, and 1,000 bootstrap replicates. The final phylogenetic trees were visualized using the plotting functions within the ape package.

#### Identification of LRR-RLK homologs in pan-eukaryotic species

To identify putative LRR-RLK homologs across diverse eukaryotic lineages, we developed a multi-step computational pipeline integrating domain-specific homology searches, structural validation, and phylogenetic analysis.

First, a reference set of full-length LRR-RLK protein sequences was compiled from published studies.^50^ Each reference sequence was analyzed using the SMART (https://smart.embl.de/)^36^ to delineate its domain architecture. Based on these annotations, the kinase domain and the leucine-rich repeat (LRR) domain were extracted separately from each LRR-RLK sequence to generate two independent query datasets.

Then, local BLAST databases were constructed for each domain dataset using the makeblastdb command. The target database was prepared by downloading the complete EukProt protein dataset (version 3) and removing all sequences from vascular plants, including angiosperms, gymnosperms, lycophytes, and ferns, resulting in a filtered set of 972 non-vascular plant species. Using blastp, we searched the kinase domain database and the LRR domain database independently against this filtered EukProt dataset with an E-value cutoff of 1e−5 and a maximum of one target sequence per query (-max_target_seqs 1). We retained only those genes that appeared in the result sets of both domain searches. These intersecting candidates were then subjected to domain architecture validation using SMART. Only sequences containing all three core domains—LRR, transmembrane helix, and kinase domain—in the correct N-terminal to C-terminal order were considered high-confidence LRR-RLK candidates and retained for subsequent phylogenetic analysis.

For phylogenetic analysis, the kinase domain sequences of these high-confidence candidates were aligned with those of representative Arabidopsis LRR-RLK subfamily members using MAFFT (default parameters). Maximum likelihood phylogenetic trees were reconstructed with IQ-TREE^37^ version 3, employing automatic model selection (-m MFP) and 100 ultrafast bootstrap replicates (-B 100). The resulting tree was visualized and annotated for subsequent evolutionary interpretation.

#### Analysis of motif evolutionary traceability

To quantitatively assess the evolutionary conservation and traceability of SSP functional motifs, we performed a systematic analysis of amino acid residue conservation across eukaryotic lineages.

First, we collected amino acid sequences of all conserved motifs of identified candidate SSP homologs from both land plants and protists. For each SSP family, we performed multiple sequence alignment using Clustal Omega (v1.2.4)^38^ with default parameters. The alignment included representative sequences from diverse land plants as well as all identified candidate protistan homologs for that particular SSP family.

Then, we defined and calculated the “Evolutionary Traceability” for each position within the conserved motifs of land plant SSPs. For every amino acid residue in the reference motifs of the 7 SSP families (excluding PIP/PIPL and PSK family because no hits in protist), we determined both Protistan-to-Land Plant (P to L) Support Frequency, and Land Plant-to-Protistan (L to P) Traceability Frequency.

Protistan-to-Land Plant Support Frequency: For a given amino acid set X at a specific position in the protistan sequences, we calculated the frequency at which the identical amino acid X occurs at the corresponding aligned position in the land plant reference sequences. This frequency (P to L) measures the conservation of a protistan residue in derived plant lineages.

Land Plant-to-Protistan Traceability Frequency: Conversely, for each amino acid Y at a position in the land plant reference motif, we calculated the frequency at which Y is found at the homologous position in the candidate protistan homologs. This frequency (L to P) quantifies the evolutionary traceability of a plant residue back to protistan ancestors.

To evaluate the inherent conservation strength of each motif position, we calculated information content (*bits*) for every residue in the SSP motifs of land plants. The seqlogo of reported SSP motifs were generated with Weblogo 3 (https://weblogo.threeplusone.com/).^39^

### Quantification and statistical analysis

All statistical analyses were performed using R version 4.4.2. To assess the association patterns of SSP family distributions across eukaryotic lineages, pairwise Pearson correlation coefficients were calculated based on binary presence/absence matrices. Specifically, we computed correlations both among the seven major eukaryotic supergroups and among the nine SSP families/subclades. The correlation matrices were generated using the R base function *cor()* and visualized with the *pheatmap* package. Significance of individual correlation coefficients was evaluated using *cor.test()*, with a significance threshold of *p* < 0.05.

## Supplemental information

Document S1. Figures S1–S3

Table S1. Sequence information of reported SSP motifs in land plants

Table S2. SSP-like sequences in protists transcriptome

Table S3. Candidate small secreted peptides (SSPs) identified from Eukprot in pan-eukaryotic species

Table S4. Candidate small secreted peptide (SSP) homologs identified from Eukprot without vascular plants

Table S5. Candidate LRR-RLK homologs identified from Eukprot without vascular plants
