# Supplementary material for: Pan-eukaryotic distribution and deep homology of plant small secreted peptides and their receptors
Source: iScience. 2026 Mar 30;29(5):115540. doi: 10.1016/j.isci.2026.115540 (PMC13091385; doi:10.1016/j.isci.2026.115540)
Supplement: Document S1. Figures S1–S3 [file mmc1.pdf]

iScience, Volume *29*

Supplemental information

Pan-eukaryotic distribution and deep homology of plant small secreted peptides and their receptors

Zhe Zhang, Songqing Yue, Fahu Yuan, and Miaomiao Zhu

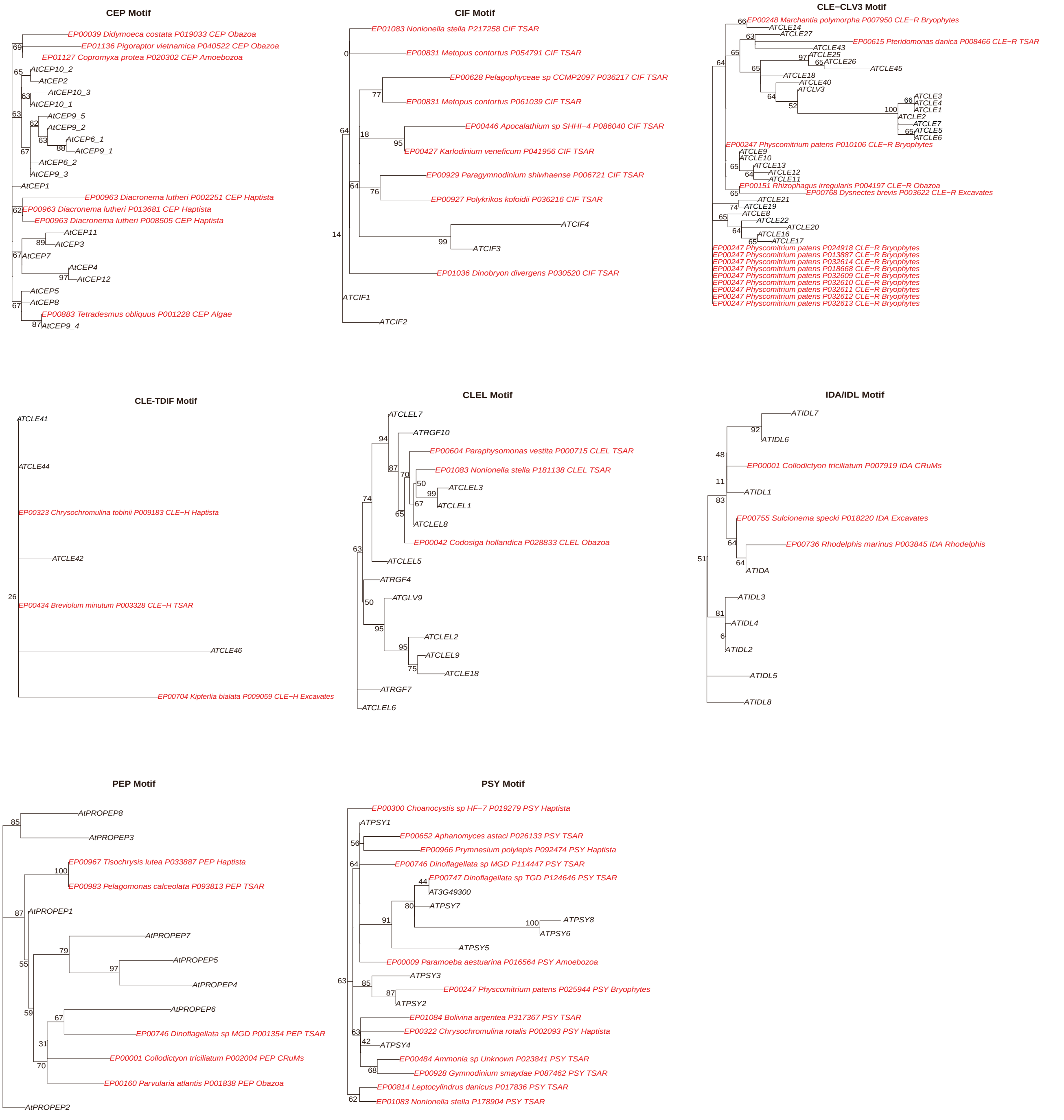

Figure S1. Maximum likelihood phylogenetic trees of each family of motifs of candidate Small Secreted Peptides (SSP) homolog.

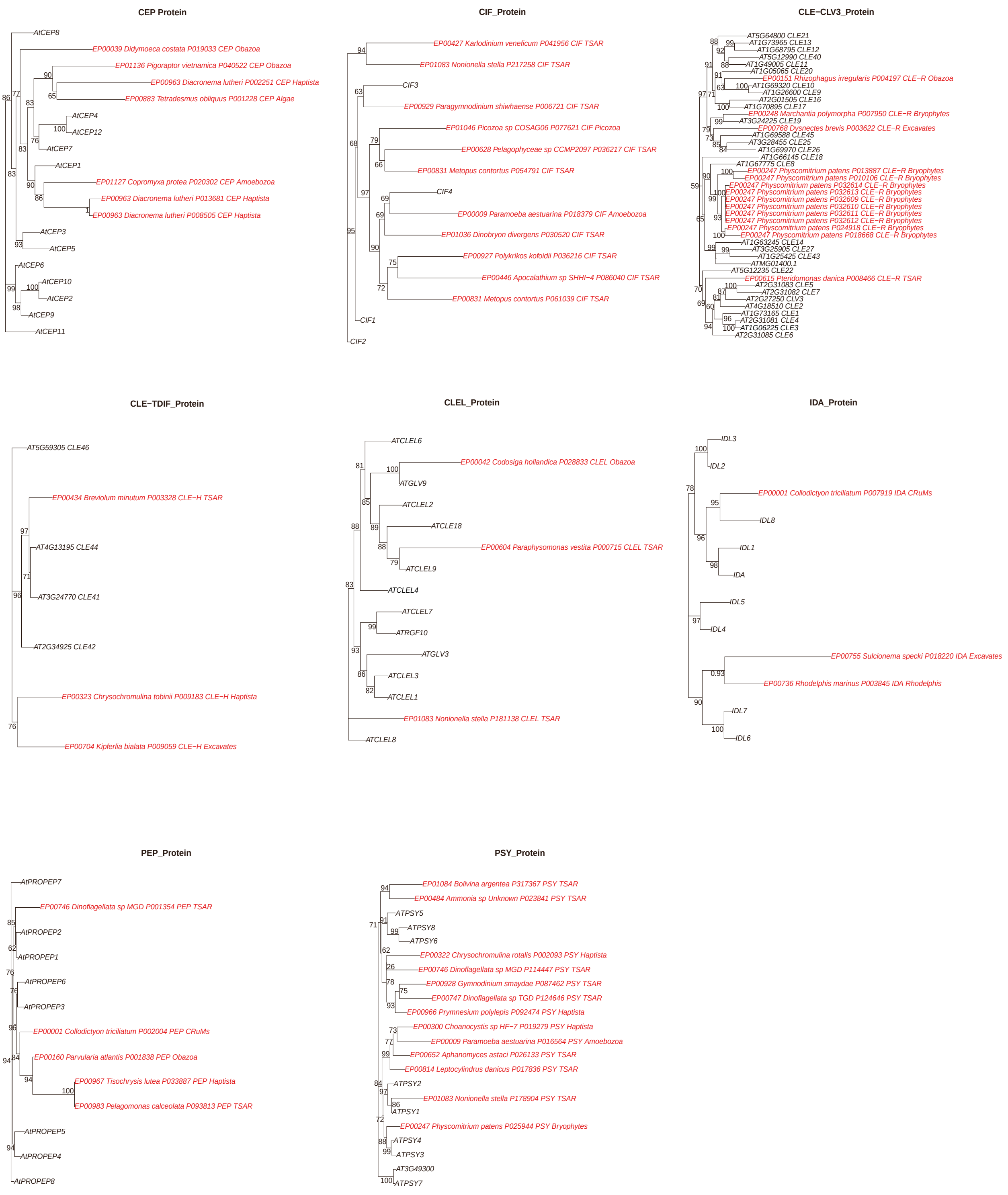

Figure S2. Maximum likelihood phylogenetic trees of full-length amino-acid sequences of candidate Small Secreted Peptides (SSP) homolog.

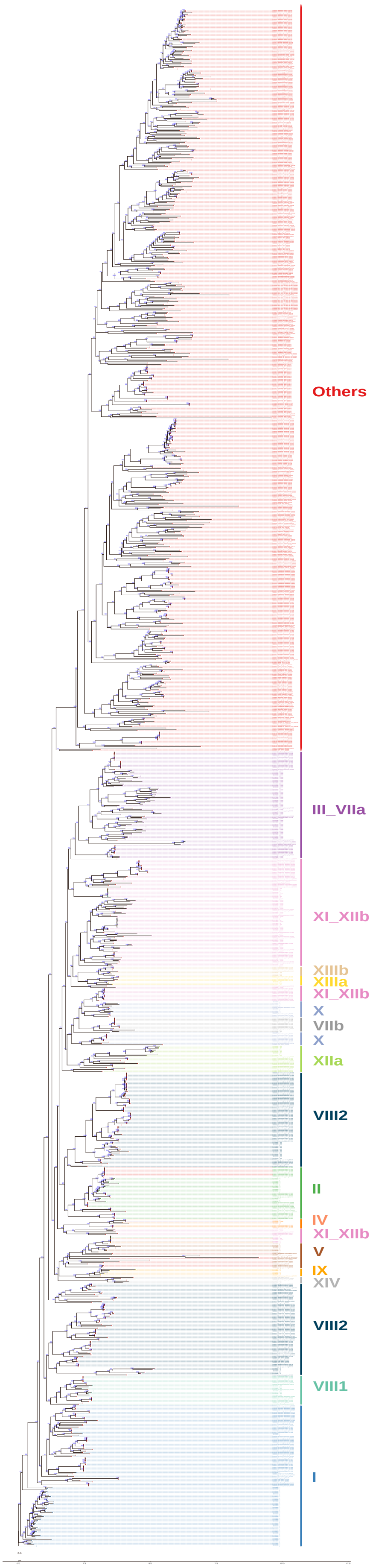

Figure S3. Maximum likelihood phylogenetic tree of LRR-RLK kinase domains.

The tree was inferred using IQ-TREE with automatic model selection and 100 ultrafast bootstrap replicates. Kinase sequences from candidate non-vascular plant homologs (EukProt) are shown together with reference Arabidopsis LRR-RLK subfamily members.
